# Taylor on Poisons

**Published:** 1848-05

**Authors:** 


### TAYLOR ON POISONS.

This very comprehensive work has also been issued by Messrs. Lea and Blanchard, in doing which they have brought the professions of law and medicine, and the public generally under obligations to them. It is safe to say that the volume of Mr. Taylor forms one of the most complete treatises on poisons extant, and that it is a work which ought to be in the hands of every physician and lawyer. It is an elaborate epitome of all that is known on the subject of poisons. The subject is one of deep interest. The crime of poisoning is unquestionably on the increase, and it is essential for the administration of justice, and for the security of society, that all the important chemical and medical facts should be collected and arranged in a form convenient for reference. And, as remarked by the author in the preface to his work, "so rapid is the advance of science, and to so great an extent is toxicology influenced by the progress of collateral sciences, that the lapse of even one year renders numerous additions and alterations necessary." We shall avail ourselves of the earliest opportunity to say more about this excellent treatise.

